# Optical surgical navigation system causes pulse oximeter malfunction

**DOI:** 10.1186/s40981-015-0007-4

**Published:** 2015-08-27

**Authors:** Masaaki Satoh, Tetsuhito Hara, Kenji Tamai, Juntaro Shiba, Kunihisa Hotta, Mamoru Takeuchi, Eiju Watanabe

**Affiliations:** 1 Department of Anesthesiology and Critical Care Medicine, Jichi Medical University, Yakushiji, Shimotsuke-City, Tochigi 329-0498 Japan; 2 Department of Neurosurgery, Jichi Medical University, Yakushiji, Shimotsuke-City, Tochigi 329-0498 Japan

**Keywords:** Neuronavigator, Saw-tooth waves, Anesthetic management, Tracking technology, Laser

## Abstract

An optical surgical navigation system is used as a navigator to facilitate surgical approaches, and pulse oximeters provide valuable information for anesthetic management. However, saw-tooth waves on the monitor of a pulse oximeter and the inability of the pulse oximeter to accurately record the saturation of a percutaneous artery were observed when a surgeon started an optical navigation system. The current case is thought to be the first report of this navigation system interfering with pulse oximetry. The causes of pulse jamming and how to manage an optical navigation system are discussed.

## Background

The Polaris spectra optical measurement system (NDI, Waterloo, Canada) is a surgical navigation system used for CT-guided stereotaxic neurosurgery. The concept of neuronavigator was introduced in 1987 [1], and optical tracking systems have been in use since 2006 [2]. A neuronavigator facilitates neurosurgical approaches.

While a pulse oximeter is an essential monitor for anesthesia management, the malfunction of a pulse oximeter was observed several times during general anesthesia management of neurosurgery, and it was finally determined that it resulted from the navigation system, and a way to manage the problem was developed.

## Case presentation

**Fig. 1:**
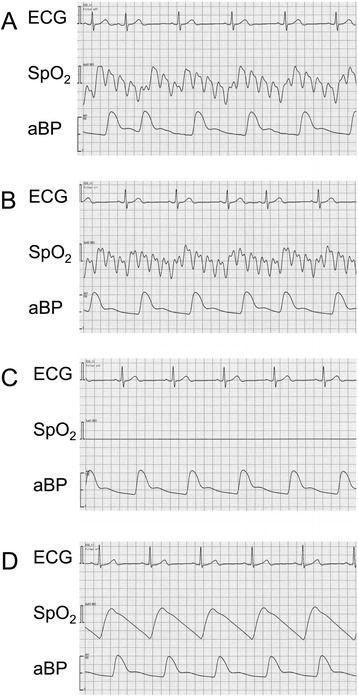
Changes in the waveform displayed on the monitor. *ECG* electrocardiograph, *SpO*_*2*_ oxyhemoglobin saturation measured by pulse oximetry, *aBP* arterial blood pressure. **a** Noise of about 10 Hz on the waveforms. **b** Noise of about 6 Hz on the waveforms. **c** Flattening of the plethysmogram. **d** Almost normal waveforms. The value of a pulse oximeter changed every beat on its display in **a** and **b**. In the case of **c**, the value of the pulse oximeter could not be displayed

A 77-year-old man with a left brain tumor was scheduled for craniotomy. He had no relevant past history. The standard monitors were used. Anesthesia was gently induced and smoothly maintained with total intravenous anesthesia. Before the operation, the surgeon set up the Polaris spectra optical measurement system, and the pulse oximeter (NIHON KOHDEN, Tokyo, Japan) showed different values for oxygen saturation (SpO_2_) and pulse rate on its display. When the pulse oximeter was affected, for example, the value of SpO_2_ changed from 99 to 94 % and then from 94 to 100 %. This phenomenon was similarly confirmed by other pulse oximeters made by a different manufacturer (MASIMO Corp, Irvine, CA). Figure 1a shows that saw-tooth waves were produced with the waves of the pulse oximeter.

The cause of this anomaly was detected by careful observation of the surgeons’ behavior. It was found that the Polaris spectra optical measurement system affected the plethysmogram. The abnormal waves varied when measured in different directions (Fig. 1b, c). Moreover, when a person passed between the Polaris spectra optical measurement system and the pulse oximeter, the phenomenon did not occur (Fig. 1d). The surgeon was then told that the Polaris spectra optical measurement system caused the pulse oximeter to malfunction, and the Polaris spectra optical measurement system was then arranged so that the surgeon was between the Polaris spectra optical measurement system and the pulse oximeter. Anesthesia was then uneventfully maintained, and the operation was successfully finished.

This appears to be the first report to indicate two crucial clinical findings related to optical surgical navigation systems: first, saw-tooth waves occur constantly during power-on, and second, that the interference can be eliminated by appropriate positioning during the operation.

**Fig. 2:**
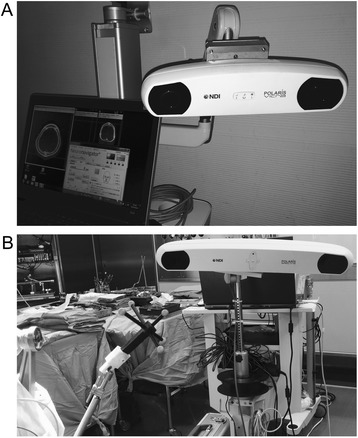
The Polaris spectra optical measurement system. **a** The main unit: a Position sensor and a PC. **b** A reference frame and the main unit

The Polaris spectra optical measurement system (Fig. 2a, b) displays positional information on the monitor using three-dimensional measurement tracking technology via a positioning laser (class 2 laser, 635-nm wavelength). Moreover, the update rate in this system is 60 Hz.

Currently available oximeters use two light-emitting diodes that emit light at wavelengths of 660 nm (red) and 940 nm (infrared). O_2_Hb and Hb have different absorption spectra at these particular wavelengths. In the red region, O_2_Hb absorbs less light than Hb, while the reverse occurs in the infrared region [3]. Sources of error in the pulse oximeter are mainly known to involve four factors: inadequate attachment, noise affecting the absorbance curve, interference with the stability of an absorbance curve, and the existence of a material attenuating the transmitted light strength.

It was thought that the Polaris spectra optical measurement system caused noise affecting the absorbance curve of the pulse oximeter. The update rate of this tracking system may directly cause flattening of the waves and a 6- to 10-Hz noise causing pulse jamming of the waveforms on the display of the pulse oximeter. In other words, the tracking system may have strongly affected the detection of the artery signal.

Moreover, the wavelength of the Polaris spectra optical measurement system is 635 nm, which is close to the 660 nm of the red region. The Polaris spectra optical measurement system could thus enhance the absorbance of Hb rather than O_2_Hb in the red region. This effect can be eliminated almost entirely by placing a person between the Polaris spectra optical measurement system and the pulse oximeter. The authors present an alternative technique. This phenomenon disappears by wrapping the sensor of the pulse oximeter in aluminum foil. When the authors explained this phenomenon to a co-author, who first used the term neuronavigator [1], he suggested aluminum foil wrapping of the pulse oximeter. He said that he had noticed this at the time of animal experiments. It is significant for anesthesiologists to be aware of this phenomenon when using the Polaris spectra optical measurement system during surgery.

## Conclusions

The Polaris spectra optical measurement system, a neuronavigator, causes pulse oximeters to malfunction by pulse jamming. This phenomenon and its effects can be normalized by positioning a person between the Polaris spectra optical measurement system and the pulse oximeter or by wrapping the sensor of the pulse oximeter in aluminum foil.

## Consent

Written informed consent was obtained from the patient for publication of this case report and any accompanying images. A copy of the written consent is available for review by the Editor-in-Chief of this journal.
